# Experience Sharing: First and Second Year of a Medical College

**DOI:** 10.31729/jnma.7085

**Published:** 2022-01-31

**Authors:** Suveksha Shaurya Shah

**Affiliations:** 1 Janaki Medical College and Teaching Hospital, Janakpur, Nepal

**Keywords:** *learning*, *medical education*, *medical student*

## Abstract

Bachelor of Medicine and Bachelor of Surgery has been a dream before it even began. This journey of half a decade is more fun than just a roller coaster ride, with numerous ups and downs and after each ride we have become stronger and prepared to embrace any challenge that life throws at us. The epic experience of medicine is not only confined to books but lies within us. Medical learning is a combination of art ,skills and knowledge for practicing medicine. Here I have shared my experience of first and second year of Bachelor of Medicine and Bachelor of Surgery.

## INTRODUCTION

Studying medicine is a dream for most of us medical students. It feels like yesterday when I had made the most important decision of my life. The basic science experience in this journey to medicine has taught me to be more hardworking, dedicated ,compassionate and has made me prepared for the next phase of clinical medicine. This is a journey to save lives and this journey is teaching us the true meaning of life as we are learning life each day and its different dimensions. The whole journey is an emotional yet self reflecting experience which plays an important role in our professional identity formation.^1^ We are learning to balance our personal professional and family life, we are learning to take care of ourselves and others, we are learning time management ,leadership and communication skills.

## WHITE COAT CEREMONY

We always had a perfect picture of being a medical doctor with white coat and stethoscope in our mind. Finally the day had come when we took our oath wearing our aprons and congratulated ourselves for being selected as medical students and our journey to become doctors had begun. White coats are not just a piece of uniform for us, it's our identity and a strong sense of responsibility. The ceremony represented the beginning of the new journey of learning medical science, taking care of people and positively impacting their health and lives for the better. Anyone who finds to save lives is amazing and we were welcomed to this amazing new world of medicine.

## CADAVERIC EXPERIENCE

The undergraduate medical course has included cadaveric dissection as a part of its anatomy teaching. It plays a significant educational roles not only in terms of consolidating our knowledge and skills on human body but also internalizes our attitude towards life and death. It is an emotional yet self reflecting experience which plays a important role in our professional identity formation.^1^ I still remember the first day of our dissection class in the anatomy department. We decided to take our lunch early before our dissection class. We prepared ourselves with our aprons ,dissecting instruments, and gloves. We entered the dissection hall, our eyes started hurting with the formalin and we went near the cadaver. We took our oath and touched the cadaver and regarded it as god of learning anatomy. After the oath our fear disappeared and we opened our Cunningham's manual of Practical Anatomy and followed the instruction of our professor. It was first time when we gave incision to a cadaver. We were going through all kinds of mixed emotions at the same time. We felt we were more close to learning human body and the journey of life and death.

## LOTS OF STUDY AND EXAMS

Medical school is about lots of hard work ,study and exams. We have to enjoy studying as joining medicine is like we have to keep on studying our whole life as a doctor ,as we need to be updated on treatment guidelines, we have to read medical journals and keep ourselves updated with new knowledge. So studying is part of our life as a medical student, and as a doctor. The journey of being a doctor is full of numerous exams. Exams are our assessment tool and if we do not prepare ourselves well from the beginning, it can be stressful specially for first year medical students as they are known to have proper study tools and techniques for study.^2^ The transition from high school to medical school can be absolutely overwhelming. The last hour of cramming is not going to work here. We should focus on understating and grasping the concepts of the topic. Exams and study are part of any student's life ,we should start to learn by prioritizing things and keeping a balance in every dimension of our life including health, family and friends.

## SLEEP, DIET AND EXERCISE

Sleep, diet and exercise are the three aspects to live a healthy life. Healthy body and healthy mind leads to a productive and efficient lifestyle. Having a balance between a balanced diet, adequate sleep and exercise will help us achieve optimal neurocognitive and psychomotor performance and will boost up capacity of grasping practical and theoretical medical learning. Medical school can be stressful and exhausting and we tend to give up our routines and affect them psychologically and psychological stress is a leading cause of insomnia and sleep disturbances. Lack of proper sleep can lead to decreased performance and depression.^3^ Having a fixed sleeping time, wake up time, exercising daily and having a balanced diet will increase our productivity. Practicing mindfulness has shown to decrease stress and depression.^4^ We are what we eat. We should eat healthy foods which are more natural rather than packed junk foods. It is said that a healthy mind resides in a healthy body. Exercise maintains our energy level all day.^5^ It provides us with the perfect kick start of the day and is proven that it improves our concentration. Therefore having a balance of sleep, a healthy diet and exercise will lead us to a better life.

## HECTIC SCHEDULE AND BURNOUTS

Medical school can be hard until we start enjoying it. Morning lectures, afternoon practical classes, assignments and lots and lots of study will be your routine . There are chances that you may burn out if you lack on managing your studies, hobbies family and friends and your break time.^6^ Being a medical student, the foremost quality that you need to develop is time management skills. Self care should be our utmost priority. There can be days of absolute breakdown, trying to keep up with the hectic schedule, trying to brush up our concepts, trying to remember everything. All of sudden, we give up and let go of our routines, crash on our bed for the whole day. The sense of laziness would feel like a sin against the big ambition we have set for ourselves. But we should not feel so. We should start learning the skill of balancing personal and professional life from very since early medical school year. We should assign a me time everyday so that we can self reflect on ourselves, find our weakness and strengths and work on it. Family and friends are our greatest support so we should spend time with and talk to them. It's okay to have bad days but we should bounce back with double the energy.

## HOSTEL LIFE AND FUN

Medical students don't have time for fun and adventure and they spend all of their time reading books is a myth. It's all about having a balance at everything you do. When it's fun time it's only fun time and when it's study time it's only study time cutting off all the distractions. Being a medical student most of us will be staying away from our home in our college hostel. Hostel became our home away from home and friends became family of that home. The depth of knowledge and experiences that we will be sharing in our classroom, laboratory, lunch at canteen, chit chat during breaks and several nights at hostel will be priceless and unforgettable. We miss home , we miss food and the love of our family. But chatting with friends on the terrace, cooking together, late night birthday parties will teach us to enjoy now and every moment we have. Hostel life teaches us how to live in a group, how to learn from each other, how to communicate, will make us strong as we are living away from our family and will teach us to create a bond ,a bond of respect, friendship and gratitude.

## WAY FORWARD

The 1^st^ and 2^nd^ year MBBS was the most overwhelming year full of learnings, challenges, opportunities, fun and lots of study. We entered into a completely new world of medicine, created a bond with books, friends, faculties, and college staff. We had a strong belief, hope ,and optimistic energy to bring positive impact within and around us. We learned life each day and we are still learning and embracing the different dimensions of life with passion, hope and possibilities. White coat ceremony made me feel like a doctor. We not only learned gross anatomy from cadaver but also learned embracing death. Staying away from home made us take care of ourselves. Having a balance between sleep, diet and exercise was so important to help us keep going. We spent most of our time in our books which took us through the ride to the world of medicine. Piercing our own fingers and taking our own blood samples made us feel the pain before giving this pain to our patients in future. Learning the names and classification of drugs and forgetting it again and again, the eosinophilic and basophilic stains sometimes took us to the galaxy. It is said that anyone who finds to save lives is amazing and we are in this amazing journey of saving lives.
